# Characterization of leukemia-specific NK cell subsets against acute lymphoblastic leukemia in children

**DOI:** 10.1186/2194-7791-1-S1-A21

**Published:** 2014-09-11

**Authors:** Florian Babor, Angela Manser, Kathrin Schönberg, Jürgen Enczmann, Arndt Borkhardt, Roland Meisel, Markus Uhrberg

**Affiliations:** Department of Pediatric Oncology, Hematology and Clinical Immunology, Center for Child and Adolescent Health, Heinrich-Heine-University, Düsseldorf, Germany; Institute for Transplantation Diagnostics and Cell Therapeutics, Heinrich-Heine-University, Duesseldorf, Germany; Department of Oncology, Hematology and Rheumatology, University Hospital Bonn, Bonn, Germany

Human natural killer (NK) cells and their antileukemic potential have been of rising interest over the last years. However, it is controversially discussed how NK cells can be best exploited for anti-leukemic therapy in the clinic. Accordingly, we performed a first detailed analysis of the NK cell repertoire specific for childhood ALL in order to investigate the participation of NK cell subsets in the killing of pediatric leukemic blasts. PBMCs from 4 healthy volunteer samples were incubated with ALL blasts of two pediatric patients. We observed a variable frequency of NK cells (0.5%-15.6%) that showed degranulation of CD107 in the presence of ALL blasts and this frequency was strongly dependent on the donor. In a next step NK cells were characterized by means of KIR2DL1, KIR2DL3 and NKG2A by 6-color flow cytometry. Analysis of CD107 mobilization revealed that especially single-KIR+ NK cells showed the highest killing ability: in single-KIR2DL1+ NK cells the frequency of CD107+ cells was 7.6% and in single-KIR2DL3+ cells 6.7%. In contrast, NK cells not expressing any of these three receptors had a reduced anti-leukemic activity, which is compatible with previous studies showing that KIR-NKG2A- NK cells are hyporesponsive. In general, KIR+NKG2A- NK cells appeared to have the highest frequency of anti-leukemic NK cells in our experiments, whereas KIR-NKG2A- consistently showed the lowest reactivity. In summary, our results showed that participation of NK cell subsets in ALL killing is dependent on respective expression levels and expression clusters of KIR and other receptors. Single KIR+ NK cell subsets appeared to be most effective against childhood ALL blasts. With further advancements in isolation and expansion techniques, development of novel characterized ALL-specific NK cells appears feasible for future donor-derived NK cell transfer as supportive treatment in childhood ALL.

